# Sensing Technologies for Autism Spectrum Disorder Screening and Intervention

**DOI:** 10.3390/s17010046

**Published:** 2016-12-27

**Authors:** John-John Cabibihan, Hifza Javed, Mohammed Aldosari, Thomas W. Frazier, Haitham Elbashir

**Affiliations:** 1 Mechanical and Industrial Engineering Department, Qatar University, Doha 2713, Qatar; 2 Biomedical Engineering Department, George Washington University, Washington, DC 20052, USA; hifzajaved1@gmail.com; 3 Center for Pediatric Neurology, Cleveland Clinic, and Case Western Reserve University, Cleveland, OH 44106, USA; ALDOSAM@ccf.org; 4 Center for Autism Pediatric Institute, Cleveland Clinic and Cleveland Clinic Lerner College of Medicine, Cleveland, OH 44195, USA; FRAZIET2@ccf.org; 5 Al Jalila Children’s Specialty Hospital, Dubai, United Arab Emirates; hoelbashir@gmail.com

**Keywords:** Autism Spectrum Disorder, eye trackers, movement trackers, electrodermal activity monitors, prosody and speech detectors, tactile sensing, social robotics, sleep quality assessment

## Abstract

This paper reviews the state-of-the-art in sensing technologies that are relevant for Autism Spectrum Disorder (ASD) screening and therapy. This disorder is characterized by difficulties in social communication, social interactions, and repetitive behaviors. It is diagnosed during the first three years of life. Early and intensive interventions have been shown to improve the developmental trajectory of the affected children. The earlier the diagnosis, the sooner the intervention therapy can begin, thus, making early diagnosis an important research goal. Technological innovations have tremendous potential to assist with early diagnosis and improve intervention programs. The need for careful and methodological evaluation of such emerging technologies becomes important in order to assist not only the therapists and clinicians in their selection of suitable tools, but to also guide the developers of the technologies in improving hardware and software. In this paper, we survey the literatures on sensing technologies for ASD and we categorize them into eye trackers, movement trackers, electrodermal activity monitors, tactile sensors, vocal prosody and speech detectors, and sleep quality assessment devices. We assess their effectiveness and study their limitations. We also examine the challenges faced by this growing field that need to be addressed before these technologies can perform up to their theoretical potential.

## 1. Introduction

Autism Spectrum Disorder (ASD) is a neurodevelopmental disorder characterized by persistent difficulties in social communication, social interaction, and restricted, repetitive patterns of behavior, interests, or activities that are present in the early developmental period [1]. Although the onset of ASD symptoms occurs during the first three years of life and the diagnosis can be reliably made as early as 24 months of life, studies have found that the mean age at ASD diagnosis ranges from 38 to 120 months, with a significant proportion of children not diagnosed until school age [2].

ASD refers to a spectrum of disorders with a range of manifestations that can occur on different degrees and in a variety of forms [3]. Since the first description of ASD by Kanner [4], emotional challenges including difficulties in understanding emotions, facial expressions, and body language continue to be emphasized as some of the main core symptoms [5]. Children with ASD tend to have difficulties in gaze and eye contact, which might be one of the earliest manifestations of their delayed development. Gaze information is important in language development and may also be important for Theory of Mind development [6], which is the cognitive capacity to attribute mental states to oneself and others. Difficulties in social interactions may be the most complex core challenge facing children with ASD [7,8]. While children on the spectrum still feel the need to form social bonds, their altered facial expressions, atypical eye contact and body language, inability to engage in a dialogue with others, difficulty in sharing imaginative play, and general withdrawn appearances may often prevent this need from becoming apparent.

To address the many challenges faced by children and adults with ASD, the role of sensing technologies becomes critical. These technologies are aimed at providing assistance in overcoming these limitations, allowing such individuals to understand and participate in the socio-emotional world around them [9]. Because ASD is not a neurodegenerative disorder, many of the core symptoms can improve as the individuals learn to cope with their environments under the right conditions. The earlier the age at which intervention can be started, the better their learning and daily function can be facilitated [10,11,12]. Hence, sensing technologies can play a key role in the screening and therapy of ASD, thus potentially improving the lives of those in the spectrum. The technologies discussed in this paper range from standalone, wearable devices to bulky equipment. These sensors measure a variety of parameters for evaluating physical, emotional, as well as environmental states that can be utilized for early diagnosis, and hence, for the improvement of the quality of life for individuals diagnosed with ASD.

By collecting specific data, these sensors may be able to acquire objective measures that can be used to identify symptoms specific to ASD. In this way, the processed data from these sensors could then be used to screen the disorder in children earlier than the current average age of diagnosis. Numerous techniques for early screening have been tested, including the detection of atypical eye gaze movements, disordered prosody, and poor quality of sleep. Once diagnosed, it is imperative that the children be imparted with skills that enable them to overcome their challenges, in order to facilitate their learning processes and improve their abilities to carry out everyday activities. Various intervention strategies have been designed by clinicians and researchers to achieve this goal. Identifying stereotypical behaviors, teaching appropriate social behavior, and facilitating emotional expression are three such strategies.

These techniques have been implemented using state-of-the art sensors, and will be discussed in detail in the next section. This paper is intended for both clinicians and technologists. Clinicians may refer to this text to assess the recent developments of clinical applications that have been tested thus far, and build on the work that has already been done to advance the usefulness of these sensors in clinical environments. Technology developers may consult this text to learn about the shortcomings of the available devices and develop the features that are needed to improve their functionality. It must be pointed out that all studies in the field, whether to assist diagnostic or therapeutic procedures, provide critical information in understanding the cause and course of this complex disorder.

## 2. Methodology

Many years of research have gone into the development of sensing devices for the improvement of the lives of individuals with special needs. The available literatures point to a large number of devices. These devices can be early screening tools, diagnostic aids, or trackers of treatment outcomes [13]. Each type of sensor has been designed with respect to a particular condition or a small subset of conditions commonly manifested by persons diagnosed with ASD. Since autism is a disorder on a wide spectrum, a variety of traits have been found to characterize the condition, and hence the same variety can be found in its sensing devices as well. These devices vary largely not just in size, but also in appearance, design, purpose, and the parameters they measure.

From the literatures on sensing technologies, the following questions were asked in order to structure the developments in this field.

1. What are the categories of sensing technologies that were intended for autism screening and intervention?
2. From the perspective of clinical utility, why are these categories important?
3. Were there experiments that showed the effectiveness of the sensors (in terms of accuracy, resolution, etc.) and their corresponding software applications?
4. Are the sensors commercially-available or are still proof-of-concepts from research laboratories?
5. What are the advantages and limitations of each sensor?

In the following sections, we present a taxonomy of sensing technologies for autism screening and interventions. We then present why each category is important and we describe works that showed novel contributions. While some of the sensors have not yet been applied in clinical environments, the majority of these have found a variety of clinical applications. Such sensors are discussed in the context of their clinical use to allow for an enhanced understanding of their roles, and to improve the prospects of finding new potential applications in the future. To assist potential users of these devices in selecting the technologies that are most suited to their requirements, the sensing technologies were compiled into a table at the Appendix. It contains a summary of their usage and lists their benefits and limitations.

## 3. A Taxonomy of Sensors for ASD Screening and Intervention

### 3.1. Eye Trackers

Individuals diagnosed with ASD have been observed to exhibit different gaze patterns that are evident as they look at socially salient stimuli such as faces [14,15,16]. Their focus is in the area around the mouth instead of the eyes [17,18,19,20,21,22]. This is a consequence of impaired social communication and interactive skills, where making eye contact is particularly challenging. The pattern of increased attention at the mouth relative to the eyes is more pronounced in natural social settings than in experimental setups [23] and has been used in diagnostic procedures to achieve the early detection of ASD [24]. It must be noted that the studies reported in this section were all conducted in laboratory settings, which is an obvious limitation for the extrapolation of the obtained results to natural social scenarios. Most of these studies were designed for early ASD screening, while some were aimed to facilitate intervention and improvement in behaviors. Driven by the availability of general use eye gaze trackers, there are more contributions in this category as compared to others. We divide this section according to desktop-based, head-mounted eye trackers, and eye tracking glasses.

#### 3.1.1. Desktop-Based Eye Trackers

One study used a desktop eye tracking device, run via the Tobii Studio package (Tobii AB, Stockholm, Sweden) to track gaze patterns of a group of 24 children with ASD (6 to 17 years old) [25]. The children were shown pictures of embedded faces and scrambled pictures with faces in them. Tobii Studio AOI (area of interest) tool was used to define the areas of interest in a picture (i.e., the face). Fixation times, which were translated to hotspot data, confirmed that children with ASD avoided looking at the faces, and in particular, the eyes. The tracker is non-invasive. It does not constrain the body or head movement, and it tracks both eyes to a rated accuracy of 0.5° and sampled at 50 Hz. An obvious concern with using desktop trackers is that the child must be facing the camera in order for the tracking to be successful. This can be difficult with children with ASD.

A more recent eye gaze tracking study by our group [24] examined 40 children with ASD and 39 children with other developmental conditions (3 to 8 years old). The aim was to determine whether remotely tracking eye gaze could yield an objective autism risk score to assist clinicians in making a diagnosis. Participants were all individuals referred for evaluation of autism after screening. The study found that aggregating gaze dwell time to social and non-social regions-of-interest strongly discriminated children with ASD from those without ASD (Area Under the Curve = 0.89, 95% confidence interval = 0.81–0.95). The paradigm was only 7 minutes and more than 90% of the participants, including those with intellectual disability, attended to the screen sufficiently to compute their risk score. These results demonstrate the viability of clinic-based remote eye gaze assessment as an objective diagnostic aide.

In another study, 31 infant siblings of children with ASD were tested at 6 months of age against a control group using a modified Still Face paradigm [26,27]. In this paradigm, the mother first interacts with the infant, then freezes and displays a neutral, expressionless face, and then resumes the interaction. The eye tracking was done using a Tobii ET-17 (Tobii AB, Stockholm, Sweden), which is a binocular infrared bright-pupil corneal-reflection videooculographic eye-tracker. It allows free motion, without incurring any delay from camera re-orientation. It also records data within a virtual box of 20 cm on each side with a precision angle of 1°, and regains signals after 100 milliseconds of interruptions, such as in blinking. Based on the obtained fixation patterns, in which the children focused more on the mother’s mouth area and avoided her gaze, 11 children were identified, 10 of which had older siblings with ASD.

**Figure 1:**
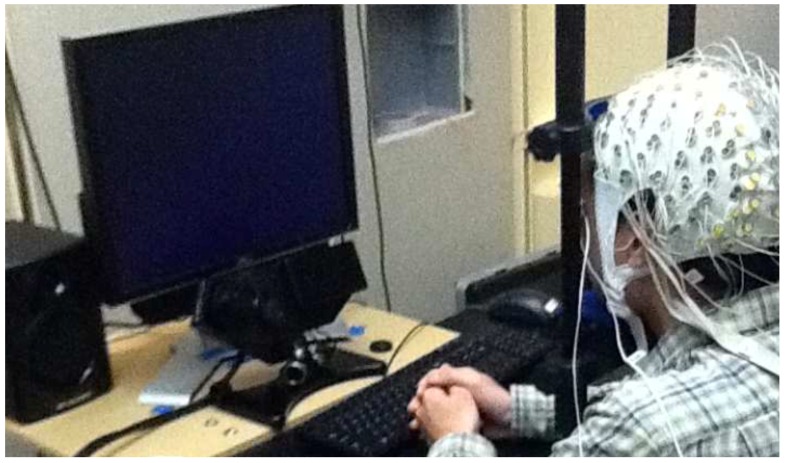
Subject with his head resting on a chin rest where eye gaze data was recorded by a desktop eye tracker (©2012 IEEE. Reprinted with permission [28]).

An initial study on a wireless electrooculography (EOG) system, accompanied by a computer game, was proposed in [28]. The system was designed to be used at home and was targeted at improving the fixation skills of an individual with ASD. The EOG activity was monitored using a signal analysis system (BioSemi B.V., Amsterdam, Netherlands) and the eye-tracking was done using the EyeLink 1000 (SR Research Ltd., Ottawa, ON, Canada; Figure 1). A game called Friends & Foes was developed to improve fixation skills. The game involves a cross appearing on a blank screen, initiating a fixation, followed by the appearance of either a friend or a foe. If the foe appears, it must be killed by a saccade from the target, but the friend must not be killed. The appearance of the friend or foe is tuned according to the collected EOG data, and the game stages become progressively difficult. The experiments were performed with a 25-year old male with corrected vision. Since improvements in fixation skills can only be seen after sufficient training, the system was designed to be portable and wireless for ease of use at home.

Another study examined the neural, behavioral, and autonomic correlates of emotional face processing in adolescents with ASD (*n* = 18) and neurotypical subjects (*n* = 20) using eye tracking and event-related potentials (ERPs) [29]. Visual stimuli comprised of faces of five females, each with a happy, sad, and fearful expression. Gaze location and pupil diameter for both eyes were monitored using a desktop-based gaze tracker (Tobii T60 monitor; Tobii AB, Stockholm, Sweden) and Clearview software (Tobii AB, Stockholm, Sweden). Eye-tracking data was sampled at 60 Hz, and three AOIs (i.e., full face, eyes, and mouth) were pre-drawn on the pictures for analysis. Three variables were eventually calculated: the duration of gaze to the eyes and mouth, proportion of time on eyes and mouth, and average pupil diameter to the eyes, mouth, and overall face. Based on the results, it was concluded that there were no differences in all three variables between both groups.

A series of studies in [30,31,32] made the participating toddlers sit down in a car seat in a dark, soundproof room in front of an LCD monitor. The session was initiated with a short cartoon video to help them settle down, followed by a calibration procedure for the eye tracker. The IView X RED eye tracker (SensoMotoric Instruments Inc., Teltow, Germany) was used at a 60 Hz sampling rate. A program was developed in MATLAB (Mathworks, Inc., Natick, MA, USA) to calculate the blink detection, perform data calibration and recalibration, and run AOI analysis. The visual stimulus shown was a 3 minute video of an actress in a setting containing four toys and a table with the ingredients for making a sandwich. The experiment consisted of four conditions: dyadic bid, joint attention, moving toys, and joint attention and moving toys. The actress’ behavior changed based on the condition. The results suggested that the subjects’ response to dyadic bids is potentially related to underlying mechanisms and must be investigated further to gain a deeper insight into the behavioral patterns that are characteristic of ASD.

Similar studies have been conducted using different commercially-available eye tracking systems [33,34,35,36,37,38,39,40,41] under similar experimental setups and procedures. As with other devices under this category (e.g., ISCAN, IView, EyeLink), the experiments usually included two groups of participants that were shown visual stimuli (i.e., still images or dynamic videos) with predefined AOIs. The subjects’ gaze patterns were tracked through a device, and the collected data was then analyzed for atypical patterns in gaze behavior.

#### 3.1.2. Head-Mounted Eye Trackers

Spezio and colleagues [22] investigated how individuals with autism utilize information as they look at others when making social judgments. To do this, they studied the relationship between subject’s gaze behavior and their fixations on regions of the face. With 9 male subjects with high-functioning autism (20–40 years old) and 10 male control subjects, the authors combined a computer vision technique called Bubbles, which reveals sparse facial information of images in an adaptive manner [42], together with head-mounted eye-tracking system (EyeLink II, SR Research Ltd., Ottawa, ON, Canada) and the accompanying EyeLink Data Viewer for the analysis of the subjects’ eye fixations. The tracker recorded data at either 250 or 500 Hz, with an accuracy of 0.5°. Experiments were run on MATLAB, using the Psychophysics [43] and Eyelink toolboxes [44]. It was found that individuals with ASD showed significant differences from the control group in terms of the features they relied upon while making emotional judgments. The subjects used very little information from the eye region and more from the mouth region.

**Figure 2:**
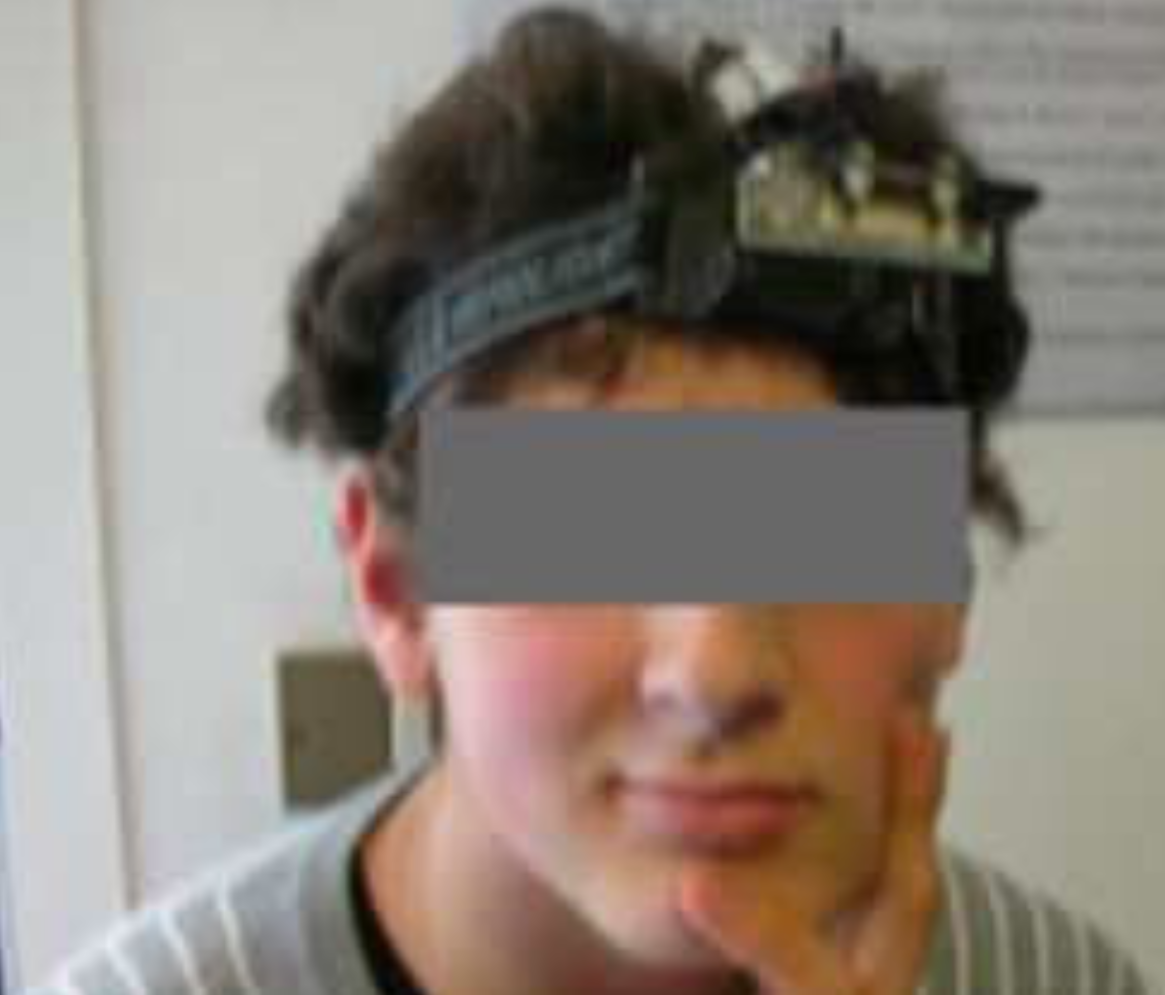
The head band of HATCAM showing a camera and mirrors to detect the eye gaze of the subject (©2010 IEEE. Reprinted with permission [45]).

The HATCAM ([45]; see Figure 2) is another head-mounted eye tracker. The HATCAM allowed mobility and helped pose the least number of distractions for a child. A rectangular mirror located on the tip of the hat reflected the image of the child’s eyes to a camera on the top of the hat to allow the capture of gaze data. The pupil’s direction with respect to the head and the orientation of the head was measured to provide eye gaze information. The calibration algorithm used images of both eyes, enabling the use of fewer calibration points and enhanced ease of interaction. However, the calculation of pupillary motion was not as accurate as compared to the results from the desktop eye trackers. In experiments conducted with four subjects (3 males, 1 female, between 7 to 20 years old), the focus of the subject’s attention towards a robot’s eyes was explored. The Childhood Autism Rating Scale (CARS) score for 8 relevant items was observed to drop or remain the same for all participants except for one.

WearCam is another monitoring device capable of measuring both the broad field of view and gaze direction from a subject’s point of view [46,47,48]. This was worn around the forehead to collect video recordings during laboratory sessions as well as free-play sessions. The videos were later analyzed to monitor the child’s focus of attention throughout the session. This wearable tracker did not restrict the child’s movement allowing him or her to interact more naturally. This mobility, however, came at the cost of accuracy of the measurements. The setup assumed that the gaze direction correlated with the head movement, which is not always the case. The camera may also not have correctly determined what the child was looking at due to its small field of view.

#### 3.1.3. Eye Tracking Glasses

**Figure 3:**
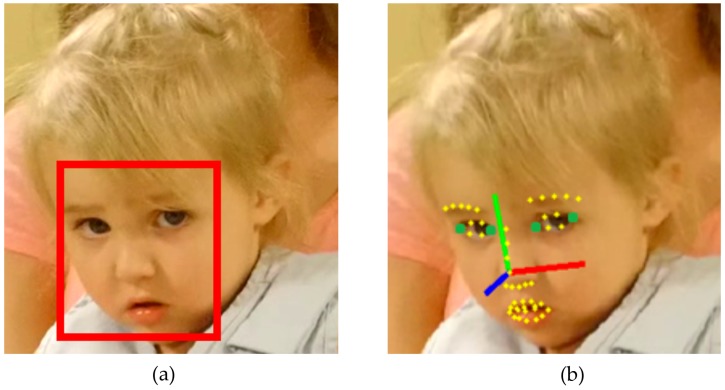
Perspective from the experimenter’s point-of-view eye tracking glasses. (**a**) Region of interest; (**b**) Head pose and eye direction overlays from a software application. (©2015 ACM. Reprinted with permission [49]).

Desktop eye trackers require a child to sit passively in front of a monitor screen. On the other hand, some children with ASD may not tolerate head mounted devices due to the contact on their skin. In situations when children need to get trained for naturalistic face-to-face interactions and eye gaze data need to be collected, both the desktop and the head-mounted eye trackers are not suitable for the task. Glasses with point-of-view cameras allow the social partner to collect videos of the child’s eyes and face and subsequent analysis of the eye gaze can be done. Ye and colleagues [49] conducted preliminary studies on dyadic social interactions between an adult and children (18–28 months old). In these experiments, a pair of point-of-view camera glasses (Pivothead, Denver, CO, USA) was worn by an adult experimenter to track the head pose and the eye gaze of a child every time the child tries to make eye contact with the adult (Figure 3).

In summary, eye trackers have been investigated to detect atypical gaze patterns for screening ASD. There are three basic categories: desktop-based, head-mounted eye trackers, and eye tracking glasses. These trackers measure the *x* and *y* coordinates of gaze fixations of subjects with respect to time. Desktop-based eye trackers are more accurate and are non-obtrusive.. However, desktop-based trackers have a limitation because the subjects must sit close to the cameras and free movement is not possible. This type of tracker is commercially-available but they are more expensive. Both the head-mounted trackers and eye tracking glasses allow more natural interactions because the subjects can stand and move around. These two are not as accurate as the desktop-based eye trackers. The head-mounted trackers are still under research while there are several eye tracking glasses that are now commercially-available. More software application developments are needed to be paired with these eye tracking glasses.

### 3.2. Movement Trackers

Children with ASD are known to engage in stereotypical behaviors, which inhibit the development of appropriate social and adaptive behaviors, and can turn into self-injurious activities [50]. A mechanism for detecting such behavior gives caregivers the opportunity for timely intervention. A case study with six children on the spectrum was conducted to observe repeated incidents of body rocking, hand flapping, and/or simultaneous body rocking and hand flapping [51,52]. The children wore sensors on their bodies in both real-world (classroom) and restricted (laboratory) settings. Each participant wore three wireless accelerometers [53], one on the left wrist, one on the right, and one around the chest, with no restriction in movement. All participants tolerated the presence of the sensors through the entire length of the observation. Each session was video recorded for offline coding using an annotation software, but real-time coding was also performed. The teachers were instructed to bring items to laboratory sessions that usually triggered stereotypical behavior. Several challenges were faced in training a classifier algorithm to automatically detect repetitive behaviors from the accelerometer readings. These included the large variations in the topography, duration, frequency, and consistency of the stereotypical movements in each participant, difficulty in generating real-time and offline annotations, and the sparsity of the occurrence of these behaviors during school hours.

Another study [54] used a custom-designed wearable sensor with 3-axis accelerometer, which could be worn at different locations on the body, in conjunction with the use of a microphone to provide contextual evidence. Four children with ASD participated in the study. Each participant showed repeated self-stimulatory behaviors such as hand flapping, body rocking, and self-harming behaviors such as face punching and leg hitting. The study was aimed at designing algorithms to automatically detect such behaviors using time-frequency and observing frequency band powers for data analysis. Linear Predictive Coding (LPC) method was used for the classification of the stereotypical and self-injurious behaviors. In principle, the LPC method minimizes the sum of the squared differences between the original signal and the estimated signal over a finite duration [55]. This method achieved a recall rate of 95.5% for self-injurious behaviors, 93.5% for flapping, and 95.5% for rocking.

**Figure 4:**
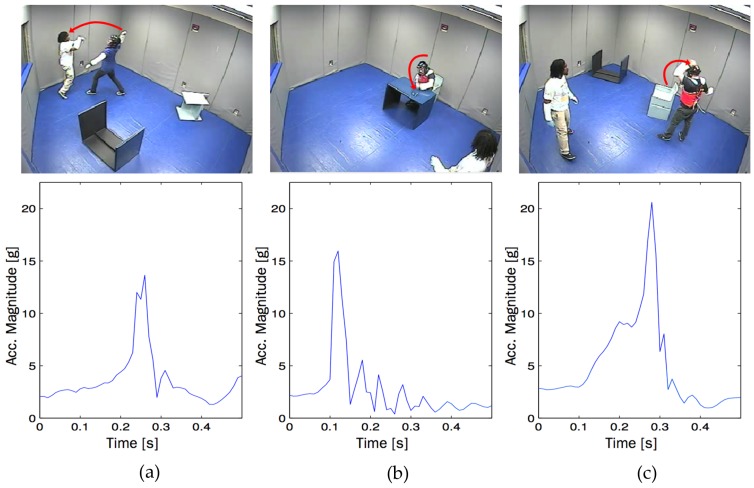
Video snapshot and acceleration readings for (**a**) aggression; (**b**) disruption; (**c**) self-injury. (©2012 ACM. Reprinted with permission [56]).

Plötz and colleagues [56] described a movement data logger for automatically assessing problem behaviors of children with ASD. The commercially-available device (AX3, Axivity, Newcastle upon Tyne, UK) consists of a micro-electromechanical systems (MEMS) 3-axis accelerometer, 16 bit microcontroller, and a single layer chip NAND flash. Combined with machine learning techniques, the authors were able to classify movement data into distinct categories of severe behavior (i.e., aggression, disruption, and self-injury; Figure 4) under three rigorous evaluations. First, sensitivity was evaluated by analysing simulated data coming from the movement data from trained clinical staff. The automated system detected severe behavior episodes with a precision of greater than 95% (recall: 41.5%) and an average accuracy of about 80% for differentiating among aggression, disruption, self-injury, and movements unrelated to problem behavior. Second, the system was tested for movements in activities of daily living from a standard dataset [57]. The automated system achieved negligible false positive results. Lastly, the system was tested for a child on the spectrum who presented problem behaviors. The system was said to have replicated the results of the assessment of an expert observer.

Two different approaches to detect stereotypical behaviors were tested in another work [58]. The first approach used a Kinect sensor (Microsoft, Inc., Redmond, WA, USA) combined with the Dynamic Time Warping (DTW) algorithm to recognize stereotypical behaviors. The other approach used the eZ430-Chronos watch (Texas Instruments, Inc., Dallas, TX, USA), which has built-in accelerometers and statistical methods for repetitive motion detection. Two separate applications were developed for each sensor, one in Windows Presentation Form (Microsoft, Inc., Redmond, WA, USA) and the other in LabVIEW (National Instruments, Corp., Dallas, TX, USA). The devices were tested with four children with ASD (10 years old on average). The number of hand flapping movements were recorded with a video camera together with the recordings from Kinect and from the watch. Using the hand flapping movements from the video recordings as the known instance of a stereotypical behavior, the Kinect system and the watch were found to have 32% error and 15% error, respectively, in detecting the hand flapping movement. The large error from the Kinect came from the position of the subject whenever the subject was too far or too near from the Kinect sensor. The watch did not have this severe problem as it was worn and acceleration data were continuously analyzed.

Majority of the earlier studies on movement trackers made use of accelerometers. Accelerometers are devices that measure the acceleration of motion of a structure. General-purpose accelerometers cost around $10 each and these have been widely used in different applications, including the detection of movements of children with ASD. With acceleration data being collected in the *x*, *y*, and *z* coordinates, these can be processed further to obtain velocity and displacement with respect to time. Furthermore, the raw signals can be converted into machine learning methods to extract meaningful measures. Accelerometers, when packaged and sold as wearable devices (with corresponding increase in price), have the benefits of being comfortable to wear and lightweight. On the contrary, any wrist-worn devices may pose danger during aggressive or self-harming behaviors. As wearable devices, the sensors are required to be in physical contact with the subjects’ bodies. Some children with ASD may not tolerate such contact in their wrist.

### 3.3. Electrodermal Activity Monitors

Wearable gadgets have become ubiquitous for their use as health tracking devices. Their utility is now extending beyond that goal to detect and assess complex behaviors in individuals diagnosed with ASD. Wearable, unobtrusive sensors allow the measurement of physiological data over extensive periods of time for continuous health monitoring. Most of the sensors discussed in this section are wrist-worn, and are capable of measuring parameters such as electrodermal activity (EDA), heart rate, and skin temperature.

EDA is used as a measure of stress levels in humans. When a person experiences stress, moisture collects under the skin as a sympathetic nervous system response [59,60,61]. The increased moisture level increases the electrical conductivity of the skin, which the skin conductance sensors utilize to determine the stress levels in a subject. However, EDA cannot communicate valence [62]. The same EDA results may be obtained when a person experiences excitement, highlighting the need for context evaluation. These sensors allow others to get an insight into the internal states of subjects with ASD, who might otherwise find it difficult to communicate their feelings. Skin temperature measurement allows the elimination of environmental factors that may cause an increase in body temperature, which can improve the integrity of the result. Tracking the motion of a subject can be used to detect, analyze and preempt repetitive, stereotypical behaviors, allowing the therapist to reinforce functional replacement behaviors. This can prevent the child from engaging in these behaviors to the exclusion of more adaptive behaviors and decreases the likelihood that certain behaviors escalate to self-injury.

Several research studies have resulted in the development of sensors with the capabilities described above. Over the years, the products have evolved in terms of comfort, wearability, ease of use, design, and the accuracy of measured data. A wearable device called iCalm was developed and it is capable of detecting heart rate, EDA, motion, and ambient temperature [63,64,65]. It took forms of a wristband, ankle band, and baby socks. The fabric is washable and electrically conductive. The sensor data was made available across devices with a wireless communication network.

The commercially-available version of the iCalm is the E4 wristband (Empatica, S.r.l., Milano, Italy). In addition to the EDA sensor, the wristband also includes a photoplethysmograph (PPG) for the heart rate, 3-axis accelerometer for movements, and an optical infrared thermometer for detecting the skin temperature. The device can be used for activities of daily living due to the device’s long battery life. It was tested for continuous data collection for 48 hours in 7 subjects [66]. The EDA variations were found to be sensitive enough to be used for young children and the elderly due to its ability to measure conductance in the 0.01–100 μS range at a default sampling rate of 4 Hz [67]. Its digital resolution is 1 digit per 900 pS.

Electrodermal activity monitors may be able to estimate a child’s internal state through physiological signals that are related to sweat rates, blood volume pulse, heart rate, and skin temperature in real-time or quasi real-time so that timely interventions can be done. Acquiring a child’s internal state could prove useful especially to some of the children with ASD who are nonverbal. Similar to the movement based trackers, the commercially-available electrodermal activity monitors are wearable and lightweight. Its main drawback is that children with self-harming tendencies might use such devices on their wrist to hurt themselves.

### 3.4. Touch Sensing

A tactile sensor is a device that can measure a contact event through touch [68,69,70,71,72,73]. Touch plays a crucial role in social communication and interactions [74,75,76,77,78,79], and may be severely affected by the challenges in tactile perception, which are commonly observed in children with ASD. This results in either hyper- or hypo-sensitivity in these children.

**Figure 5:**
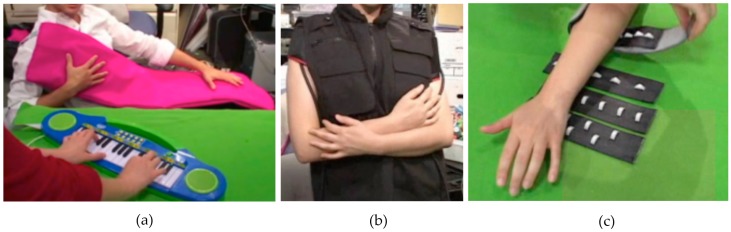
Various experimental haptic interfaces for therapy: (**a**) Touch Me simulates touch; (**b**) Squeeze Me simulates hugs; (**c**) Hurt Me induces controlled pain. (©2009 ACM. Reprinted with permission [80]).

One study attempted to convey emotions to individuals with ASD using the sense of touch [80]. Three haptic devices were developed: Touch Me (Figure 5a), Squeeze Me (Figure 5b) and Hurt me (Figure 5c). These use vibrotactile, pneumatic, and heat pump actuation. Touch Me can be used on the arms, legs and chest areas. It has an enclosed vibrotactile motor array that be remotely activated to control the intensity and location of the actuation. Squeeze Me is a vest designed to simulate hugs on the press of a button. It has pneumatic chambers around the shoulders, chest and back, which are temporarily inflated by an embedded air compressor to provide distributed pressure. Safety features to prevent over-inflation have been integrated in the design. Hurt Me is a pneumatic bracelet, which generates controlled pain within safety limits by inflating a pressure bladder studded with plastic teeth. This is designed for the subset of population with ASD that has self-harming tendencies.

Another contribution to this domain is a vibrotactile gamepad that was designed to provide emotional feedback to the user [81]. It has 16 tactile sensors on its right half, each of which is made of a conductive film. In principle, the user is required to wear a conductive bracelet in order to close the electrical loop for gamepad function. The idea is to let the participants to play video games on a computer using the gamepad, which would be triggered by commands sent from the computer. Based on the events in the game, different vibrotactile patterns would be generated whereby each pattern is linked to the other in order to create a vibrotactile language. This would be beneficial because it creates an intuitive haptic feedback at par with visual cues. In an initial user study, nine users (22 to 25 years old) were asked to create vibrotactile patterns for six basic emotions: anger, fear, disgust, happiness, sadness, and surprise, by varying the frequency, amplitude, and duration of actuator activation. These were accompanied by suitable visual and audio stimuli to convey emotions. In the second user study, nine participants (10 to 16 years old) took part. Three of the subjects were hypersensitive, two were hyposensitive, and the remaining had no particular disorders. They were presented with the gamepad to play the specially designed video games. The games were integrated with the most intuitive patterns created for each emotion from the first user study. It was found that 50% of the participants relied on the vibrotactile patterns to interpret and memorize the emotions. Enhanced attentional focus and emotional understanding were also observed among the participants.

**Figure 6:**
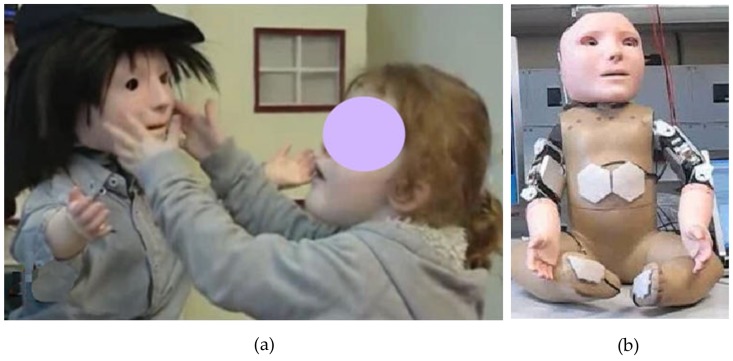
Interactions between a child and a touch-sensitive social robot. (**a**) Games with tactile contact interaction; (**b**) Hexagon-shaped tactile skin patches on the robot KASPAR. (With kind permission from Springer Science + Business Media, adapted from [90]).

Social robots are being used as assistive tools for autism interventions and as learning companions [82,83,84,85,86,87,88,89]. One of those is KASPAR, a child-sized humanoid robot [90,91] that is being used as a therapeutic tool for children with ASD. The interactions were designed to encourage children to touch the robot to broaden the scope of possible interactive scenarios (Figure 6a). For this purpose, KASPAR was equipped with tactile sensors on its cheeks, torso, both arms, palms and at the back of the hands and feet [92] (Figure 6b). The sensors, working on capacitive sensing principle, have layers of foam over them to hide them and to distribute the contact pressures [93]. In [94], the authors tested whether appropriate physical social interactions can be taught to children with ASD. Children with ASD need to modulate the force they use in touching others. Eight boys diagnosed with ASD (six to nine years old) participated in the study that compared the gentle touches and the harsh touches of the children to the robot and to the experimenter. Results showed that there were significant differences between1 the gentle and harsh touches on the robot and on the experimenter. The sum of the gentle touches applied by children to the robot was 8.5 times higher than the harsh touches. When the children touched the experimenter, the gentle touches were 23.6 times higher than the harsh touches. It was demonstrated that a robot equipped with touch sensors can help therapists train social feedback to children with ASD for them to apply the appropriate contact forces to others.

The tactile sensors used in autism research are sensors that generally detect contact pressure. These sensors are mounted on various interfaces. These interfaces include wearable systems (e.g., jackets, wristbands or foot wear), gamepads, and social robots. Once tactile contact has been detected, the wearables and gamepads then provide vibration feedback. The robots, on the other hand, can speak or react based on the intensity of contact on the robots. Taken as a whole, systems that provide haptic feedback have been used as intervention tools to help improve a child’s tolerance to physical contact. Through the tactile sensors, robots can get the necessary signals so that it can react to the touches from a child. The next generation smart interfaces with tactile sensors will enable clinicians to teach children appropriate social skills that they can use to repeat the social touching interaction with their family members and friends. Future designs of these sensors should be robust to withstand severe touches.

### 3.5. Prosody and Speech Detection

Several studies have identified vocal differences in children diagnosed with ASD as compared to neurotypical children [95]. The reported prosodic difficulties include the production of monotonic intonation, abnormal stress patterns, deficiencies in voice quality, and uncontrolled loudness. Studies on prosodic difficulties encompass both prosodic production [96] and perception [97]. The difficulties in affective prosody recognition in children with ASD are attributed to the more generic emotion recognition challenges that is characteristic of the autism spectrum [98].

Devices have been built to detect distinctive speech patterns to be used as early ASD screening tools for toddlers. Encouraged by the growing evidence that early markers of ASD may be present before the age of two years [99], a study was conducted to detect acoustic differences in pre-verbal vocalizations. Using available data from another study called the Canadian Infant Sibling Study [100], two groups of children comprised of controls (low-risk) and younger siblings of probands with ASD (high-risk) were analyzed throughout infancy [101]. Acoustic-prosodic features were extracted from speech recordings of both groups using the VoiceSauce speech recognition software [102], accompanied by an energy threshold strategy, which was devised to mark the start and end of vocalizations. Pattern recognition was then used to identify salient features that were used to classify the subjects as neurotypical or those with ASD.

In another study [103], a multi-stage Bayesian classifier was developed to distinguish between the five categories of prosodic speech, namely prohibition, approval, soothing, attentional bids, and neutral utterances. From vocal samples taken from typical male and female adults, the classifier was able to accurately identify the categories 75% of the time. This is in comparison to human judges, who did this with a 90% accuracy rate [104]. The classifier does this by separating the low-energy prosodic categories (neutral and soothing) from the high-energy prosodic categories (approval, attention, and prohibition). The classifier, however, has not yet reached a stage where it can be clinically used as a diagnostic tool.

**Figure 7:**
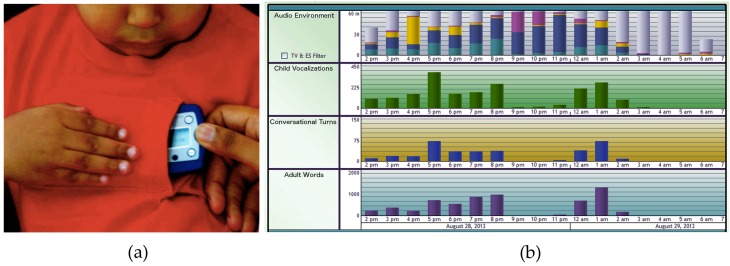
The Language ENvironment Analysis (LENA) device. (**a**) Custom-designed clothing with a pocket to insert the recorder; (**b**) Software interface of LENA showing the audio environment, child’s vocalizations, conversational turns and adult words throughout the day. Images courtesy of Dr. M. Aldosari, Cleveland Clinic.

Language ENvironment Analysis (LENA, LENA Research Foundation, Boulder, CO, USA) is a voice-recognition system that monitors how children with ASD vocalize and engage verbally with others. It is being used by researchers and clinicians as an early ASD screening and treatment tracking tool. The product comes with custom-designed clothing with a pocket to insert the LENA recorder (Figure 7). It is used to collect, manage, and analyze audio recordings of children from 2 to 48 months of age. The analytics provide the count and percentile data on speech-language measurements such as the number of words spoken by adults to and around the child, external noise such as TV sounds, adult-child conversational interaction, and child vocalizations. One study [105] tracked sounds produced by 26 children with ASD (between 16 and 48 months old) within a 12-hour period. It was found that children with ASD have 26% fewer back-and-forth vocal interactions with adults than neurotypical children, and the interactions were about four seconds shorter. It was also found that when children with ASD vocalize, it is often not directed at anyone. A limitation of this study is that the analysis made no distinction between simple and complex utterances, which could have provided improved results.

In summary, vocal prosody and speech detectors can be potentially used to detect atypical vocal patterns for the early diagnoses of ASD in children. There were two approaches used so far and both require the processing of audio recordings into meaningful information. The first type extracts the prosodic features, such as pitch and energy, of a child’s utterances. From these features, the classifier can discriminate according to soothing, approval, prohibition, attentional bids, and neutral speech. The second type is a voice recorder with Language Environment Analysis (LENA). This method counts the child’s vocalizations, conversational turns, and the words spoken by the adults around the child throughout the day. These two types of prosody and speech detectors have shown limited success and need further development for them to be used in clinical applications.

### 3.6. Sleep Quality Assessment Detection

About 50%–80% of the children diagnosed with ASD have poor quality sleep as compared to 9%–50% of neurotypical children [106,107]. Although sleep disorders are not part of the diagnostic criteria, they occur commonly enough to be regarded as a characteristic of the autism phenotype [108,109]. Multiple sleep detection methods have been developed to assess the sleep quality for the diagnosis of sleep disorders and to evaluate the effectiveness of interventions.

#### 3.6.1. Polysomnography

Polysomnography measures multiple neurophysiological and cardiorespiratory parameters to provide an insight into activities that occur during sleep, such as eye movements, muscle activity, and oxygen saturation levels [110]. This procedure allows the identification of narcolepsy, hallucinations, sleep paralysis, apnea, and sleep-related problems that are not otherwise detectable. While this method may provide the most valuable data for sleep quality evaluation, it is often not tolerable by the children with ASD [111]. In addition, most of the studies conducted so far cannot be deemed conclusive due to the small sample size of suitable participants and limited generalizability [112,113,114]. The cost of the procedure and the prerequisite travel to the laboratory also adds to the disadvantages. The obtained results might not reflect accurate results since the child’s sleep pattern can be easily affected by the unfamiliar surroundings of the laboratory [115,116]. Sensors that measure the required parameters also need to be in constant physical contact with the patient’s body, making it more impractical.

#### 3.6.2. Actigraphy

Another method reported in the literatures is actigraphy. This method uses watch-like devices (i.e. actigraph) on the wrist or ankle to detect nighttime movement of the limbs in order to determine sleep-wake cycles [117,118,119]. Parents have reported that children with ASD were observed to lay awake quietly, without movement [120]. Actigraphy falls short in measuring wakefulness in such cases due to the absence of measurable motion in the participants [121,122]. It was shown that actigraphy accurately detected sleep 92% of the time but only detected wakefulness only 48% of the time [123]. This can be attributed to the actigraphy’s inability to detect wakefulness in children who were quietly lying awake. Though it is less obtrusive than polysomnography, between 10% and 33% of the children with ASD could not tolerate actigraphy [124,125].

#### 3.6.3. Video Monitoring

Video monitoring devices have been proposed as a less obtrusive method to monitor subjects as they sleep [126]. Upon reviewing the video recording, the observers can determine the sleep-wake states, percentage of time spent sleeping, and the time spent in a state of quiet wakefulness. This method has the advantage of being better tolerated by the subjects. It is also capable to measure the motionless states of wakefulness that may go unnoticed from an actigraph [127]. Sitnick and colleagues [127] reported that video monitoring and actigraphy were significantly correlated on total sleep time, sleep latency, and waking after sleep onset. The correlations ranged from 0.26 for the number of night awakenings to 0.96 for sleep onset time. As compared to polysomnography, video monitoring is less intrusive but it can make the parents and the children feel uncomfortable with it. We did not find other available studies that compared the accuracy of video monitoring to other sleep assessment methods.

#### 3.6.4. Ballistocardiography

**Figure 8:**
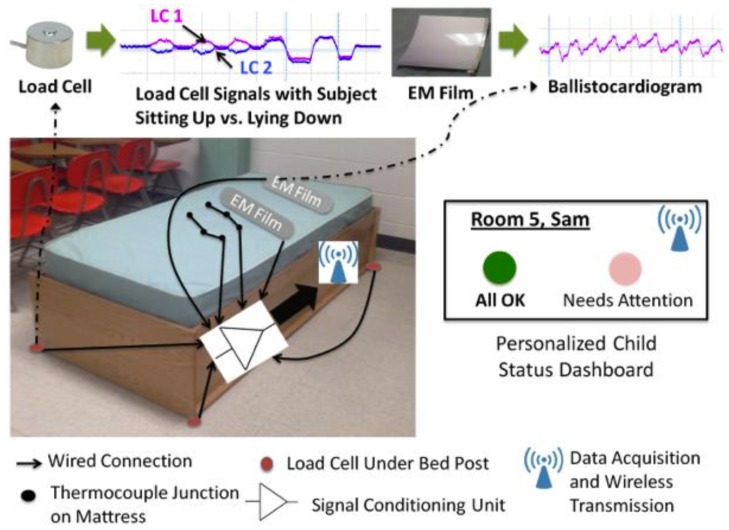
The bed sensor suite and the residential dashboard interface indicating the real-time status of the child on the bed. Sample data shows movements and ballistocardiogram (BCG) data (©2014 IEEE. Reprinted with permission [128]).

Unobtrusive methods were employed at residential and educational facilities to evaluate behaviors of subjects in bed for their sleep quality and movements [128,129]. Such a method measures the ballistocardiogram (BCG), which is an evaluation of a subject’s ballistic forces, enabling the determination of both cardiac and respiratory data. Prakash et al. [128] explored two possible methods for BCG measurement (Figure 8). One is the use of electromechanical force films, which were placed on top of the child’s mattress and under a layer of memory foam to prevent perception of the films and to improve comfort. The other technique involved the use of six load cells. These were placed under the frame of the bed in order to determine whether or not the child is lying on the bed, to characterize the activity level of the child, and to measure his or her BCG. The bed frames were also supplemented with devices that can measure the mattress temperature with thermocouple sensor grids to alert caregivers of bedwetting incidents. An acquisition module collected data from all the sensors, uploaded them through a wired or wireless link to a central data collection instrument, and displayed the summarized results on a dedicated dashboard for every subject. When a subject was asked to lie on the bed in a supine position, the preliminary results showed that the coefficient of correlation of the BCG data to electrocardiogram data was 0.989. This demonstrates the ability of the BCG to detect the subject’s heart beat and estimate heart beat intervals.

Detecting the quality of sleep could be a useful measure to tailor the intervention activities of the child the following day. We found four types of sensing methods for sleep monitoring. These are polysomnography, actigraphy, video monitoring, and ballistocardiography. Polysomnography records neurophysiological and cardiorespiratory signals to determine eye movements, muscle activity, and blood oxygen levels. Among all the available methods, this has the highest level of accuracy. Its main drawback is that the electrical wirings that connect the sensors to the instrumentation system are obtrusive even for the general population. In addition, the sensors are required to be in constant physical contact with the subjects as they sleep. An actigraphy system measures movements during sleep through accelerometers. This type is less obtrusive as compared to a polysomnography system. Its main limitation is that the data will not show whether a child is awake or is just lying motionless on the bed. A video-monitoring system, like any general video recording system, requires observers to re-play the video and watch subjects as they sleep so that analysis can be done. This is a non-obtrusive way to detect sleep and it addresses the limitations of the actigraphy method because the video will show whether the subject is awake or not. However, video monitoring systems require that an observer watches the subject’s video for the whole duration that the subject is sleeping. The main disadvantage is privacy. The last method is ballistocardiography. This method can measure the heart rate, respiratory rate, movements, breathing, and bedwetting incidents. This method is also non-obtrusive and current developments allow wireless transfer of data. Its main limitation is the retrofitting of the bed frames to accommodate the sensors. With this type of detection, sleep can be monitored even without video recordings. Hence, there can be less concerns on privacy.

## 4. Discussion and Conclusions

A trend in the general health care industry has been to move towards preventive measures and outpatient monitoring, in order to continuously monitor health and track progress in all stages of life and disease. This has caused health monitoring to become part of the fabric of life, making general health trackers available ubiquitously.

However, developing such technologies for ASD is especially challenging. This is in part, due to the exceptional nature of the users. The diverse nature of autism spectrum disorder presents challenges on a number of fronts that must be tackled simultaneously in order for the technology to be effective. Many of the sensors described have not moved beyond the initial phase of feasibility for use with children, and some have not been rigorously evaluated. There appears to be three separate issues that increases the difficulty. The first is the sensing technologies themselves, the second is the diverse nature of the subjects, and the third is the nature of the experiments that test new types of sensor technologies on diverse experimental subjects. Nonetheless, these reasons should not discourage the development of objective measures that need to be collected, analyzed, and reported to all the stakeholders.

Fundamental issues have to be first addressed before these sensors can be used for screening and interventions. Empirical evidence must be presented that shows a sensor’s repeatability, robustness, and demonstrate results that are comparable with the accepted measurement standards. Furthermore, the sensing devices are required to be non-invasive, allowing children with ASD to focus entirely on the interaction without distractions. They must also be portable, lightweight, affordable, and user friendly, in order to be used easily at home where the child is expected to interact most naturally. The state of the art in sensing technologies has not yet enabled all these requirements to be met. Most observations in the studies discussed in this paper have been conducted in restricted laboratory environments, where the subjects’ activities are controlled. Hence, the results have to be taken in the proper context.

On-demand data analytics from the sensor system is another challenge. This creates opportunities for therapists and clinicians to engage in real-time intervention once an observation of interest is made. While some of the available technologies provide real-time analysis of the tracked data, many still fall short. Lastly, privacy and security of the tracked data also need to be ensured for all the participating individuals as it is communicated to multiple devices over wireless networks. Some of the sensors mentioned in this paper are still in the preliminary stages of development, and have not yet been tested clinically. Because the potential of a sensing device cannot be accurately determined until it has been tested in a clinical environment, such devices cannot yet be deemed fully reliable. All the devices that have been applied clinically have been discussed in the context of their respective studies in the current paper in order to improve the understanding of their uses and capabilities.

In some cases, a technology is unable to deliver up to its potential, not due to the hardware but due to the inefficiency of the accompanied algorithms, as in the case of classifiers for repetitive behavior detection. Therefore, equal emphasis needs to be placed on the improvement of all aspects of a tracking technology. The nature of the sensors makes the tracked data very sensitive to experimental and systematic errors [56], often causing the collected data to be discarded due to unreliability [130]. Efforts to reduce such inaccuracies can significantly improve the performance and potential of the overall technology.

It is evident that significant improvements are required on a number of fronts in this domain before these devices can reach a high standard of acceptability and reliability, which are needed for large-scale adoption. However, it is important to mention that current diagnostic and treatment tracking approaches are highly subjective and rely heavily on the subjective impressions of clinicians and parents. They are also costly and require substantial training to administer. Sensing technologies allow for the extraction of objective measures that can address some of these shortcomings, resulting in less costly and time-intensive evaluations, which can increase the efficiency and acceptability of the evaluations.

The research community is shaping the future standards, metrics, and practices in the domain of sensing technologies for ASD. While sensing technologies have become increasingly reliable and usable, it is clear from our analysis that the room for improvement remains large. Therefore, much work still needs to be done before these devices can fully reach their theoretical potential, and can become capable of replacing traditional diagnostic and intervention mechanisms for ASD. It is important to note that each research effort in the field is crucial, and is a step towards improving our understanding of the complex and diverse nature of autism spectrum disorders.

## Appendix A. Summary of Sensing Technologies for ASD Screening and Intervention

**Table:**

Sensor Category	Purpose	Type	Measured Quantity	Benefits	Limitations
Eye trackers	To detect atypical eye gaze patterns for early screening	Desktop-based eye trackers	Timestamp, *x* and *y* coordinates of gaze fixations, distance from the display or stimulus	More accurate than head-mounted devices or the glasses Non-obtrusive	Subject must face the camera, free motion not possible in clinical settings Can be expensive Requires calibration for every subject
		Head-mounted eye trackers	Timestamp, *x* and *y* coordinates of gaze fixations, distance from the display or stimulus	Mobility allows for more natural interactions Requires fewer calibration points (for HATCAM)	Pupillary motion calculation is not very accurate (for HATCAM) Does not account for head movement, compromising accuracy (for WearCam) Some designs are obtrusive
		Eye tracking glasses	Timestamp, *x* and *y* coordinates of gaze fixations, distance from the display or stimulus	Mobility allows for more natural interactions First person point-of-view	Less accurate than desktop-based devices More software applications need to be developed
Movement trackers	To detect stereotypical movements for timely intervention	Wrist wear, worn on the chest, desktop	Acceleration, velocity or displacement in *x*, *y*, and *z* coordinates	Comfortable to wear, small, light High level of accuracy Easy to use	Wrist-worn devices may pose danger during aggressive/self-harming behaviors Requires physical contact with the subject’s body Variations in movement duration and frequency movements by participants result in experimental challenges
Electrodermal activity monitors	To estimate the subject’s internal state through physiological data for timely intervention	Wrist wear	Electrodermal activity, blood volume pulse, heart rate, skin temperature	Comfortable to wear, small, lightweight High accuracy Easy to use Long battery life	Requires physical contact with the subject’s body to track physiological signals Wrist-worn devices may pose danger during aggressive or self-harming behaviors Electrodermal activity cannot determine emotion valency
Tactile sensors	To simulate touch and hugs, and to induce controlled pain (for subjects with self-harming tendencies)	Worn on the wrist, chest, or leg	Contact pressure, then provides tactile feedback	Improves tolerance to physical contact	Requires constant physical contact which can be troublesome May pose danger during aggressive/self-harming behaviors
	To provide emotional feedback while playing games and to evaluate the accuracy of the subjects’ responses	Vibrotactile gamepad	Contact pressure, then provides vibrotactile feedback	Improves tolerance to physical contact	Requires constant physical contact which can be troublesome May pose danger during aggressive/self-harming behaviors
		Touch sensors on social robots	Contact pressure, then classifies the contact behavior to provide appropriate feedback	Reacts with verbal and visual responses to tactile interactions from subjects to teach appropriate social skills Robot imitates natural human interactions due to the touch sensing and feedback	May not always be able to classify detected tactile behavior correctly Sensors and the robot hardware may not be robust during a child’s meltdown
Vocal prosody and speech detectors	To detect atypical vocal patterns for early diagnosis	Voice recording and pattern recognition	Detects prohibition, approval, soothing, attentional bids and neutral utterances	Classifies children as atypical or typical, which is a valuable final outcome Can detect and distinguish between a variety of vocal characteristics	Needs to be developed further before it can be used for clinical applications
		Voice recording and LENA (Language ENvironment Analysis) device	Counts the number of words spoken by adults to and around the child, adult-child conversational interactions and child vocalizations	Comes with custom-designed clothing Small and portable Provides acceptable data analytics reports	Suitability for clinical applications not yet proven
Sleep quality assessment devices	To get an early indication of ASD since poor sleep quality may serve as a possible indicator	Poly-somnography	Neurophysiological and cardiorespiratory parameters to determine eye-movements, muscle activity and oxygen	High level of accuracy	Obtrusive and not tolerated for long by most subjects Limited to be used inside a laboratory Expensive Sensors required to be in constant physical contact with the subject’s body
		Actigraphy	Movement data through accelerometer readings	Less obtrusive than polysomnography	Children may be awake but motionless, which will go undetected Difficult to wear the actigraph through the length of the experiment
		Video-monitoring devices	Video data	Unobtrusive Can help detect motionless wakefulness unlike actigraphy	Requires re-playing long video recordings Privacy issues
		Ballisto-cardiography	Heart rate, respiratory rate, activity detection, bedwetting incidents	Unobtrusive Measured data transferred wirelessly to a console for visualisation Reliable data measurement	Requires custom-made bed frames equipped with the apparatus
